# Vision-related quality of life among released from treatment cases of leprosy evaluated with NEI-VFQ-25: a cross-sectional study

**DOI:** 10.1186/s12886-023-03146-2

**Published:** 2023-10-02

**Authors:** Yunia Irawati, Gitalisa Andayani, Tri Rahayu, Hindun Zakiyah, Dewinta Retno Kurniawardhani, Carennia Paramita, Anna Puspitasari Bani, Hisar Daniel, Made Susiyanti, Yeni Dwi Lestari, Dewi Friska, Sri Linuwih Menaldi, Melinda Harini

**Affiliations:** 1 Department of Ophthalmology, Faculty of Medicine, Universitas Indonesia, Dr. Cipto Mangunkusumo Hospital, Jakarta, Indonesia; 2 Department of Community Medicine, Faculty of Medicine, Universitas Indonesia, Jakarta, Indonesia; 3 Department of Dermatology and Venereology, Faculty of Medicine, Universitas Indonesia, Dr.Cipto Mangunkusumo Hospital, Jakarta, Indonesia; 4 Department of Rehabilitation Medicine, Faculty of Medicine, Universitas Indonesia, Dr. Cipto Mangunkusumo Hospital, Jakarta, Indonesia

**Keywords:** Leprosy, Quality of life, Disability, Visual functioning questionnaire, Visual impairment

## Abstract

**Background:**

People with leprosy who have been declared Release From Treatment (RFT) are often not aware of the leprosy sequelae possibility which can decrease their quality of life. This could be because they have been adapting for a long time hence they do not feel the need to see physicians. This study seeks to compare the results of Vision-Related Quality of Life (VR-QoL) among RFT persons based on the National Eye Institute Visual Functioning Questionnaire–25 (NEI-VFQ-25**)** and WHO grading disability based on physical examination.

**Methods:**

A cross-sectional study of 325 RFT subjects from leprosy communities (Singkawang, West Kalimantan and Tangerang, Banten) was conducted between 2018 and 2019. We used the NEI-VFQ-25 questionnaire that had been validated and translated into Indonesian and distributed to the leprosy population. Relationships and comparisons among variables were evaluated using Kruskal–Wallis and Mann–Whitney tests.

**Results:**

There were three main results: The median composite score of VR-QoL for WHO grade 0, 1, and 2 disabilities has decreased by 13%, 25.5%, and 30% of the maximum value, respectively. Of the total, eleven subscales were statistically significant between WHO grading disability and VR-QoL based on the NEI-VFQ-25 (*p* < 0.05). The comparison between grade 0 and grade 2 disability in all subscales was statistically significant (*p* < 0.05).

**Conclusions:**

The grade of disability is related to their VR-QoL assessment using the NEI-VFQ-25 questionnaire. Thus, it can be used as an initial screening in primary healthcare settings to increase awareness of disability before a thorough physical examination.

## Background

Leprosy is an infectious disease caused by *Mycobacterium leprae*, which primarily alters the skin, peripheral nerves, eyes, and other mucosae [1]. Delayed detection and inappropriate treatment can cause the disease to progress badly. The development of the damage can occur before, during, or after treatment. The high incidence and complications will lead to several causes such as physical impairment due to nerve damage and psychosocial impacts [2, 3].

The incidence of leprosy is declining, but not at the expected rate as reported by 161 countries in 2019 that a total of 202,185 new leprosy cases were discovered worldwide [4]. Brazil, India, and Indonesia were at the top of the list with more than 10,000 cases each [5]. In 2017, Indonesia announced 15,910 new cases of leprosy (6.1 / 100,000 populations) with 86.12% of cases including *MultiBacillary* (MB) type, and 61.99% of new leprosy patients were male [6]. Data from the Indonesian Ministry of Health in April 2021 showed that there were still 7 provinces that have not been eliminated from leprosy [7]. The World Health Organization (WHO) recently initiated a new global strategy for leprosy 2021–2030 entitled "Towards Zero Leprosy". The focus of attention on leprosy is now shifting more towards the prevention of disability through early case findings [8].

The WHO definition of Quality of Life (QoL) is a personal perception of their position in life, the culture and value systems in which they live, and their goals, standards, expectations, and concerns [9]. Leprosy has a great risk of permanent and progressive physical disability, thus causing an impact on a patient’s QoL. Studies conducted in India, Bangladesh, and Brazil demonstrated that the QoL of leprosy patients was worse than that of the general population [10, 11]. Although various efforts have been made to minimize the progression of disability in people with leprosy, there are still numbers of former leprosy patients who have recovered from the disease with residual defects that affect their limbs and eyes [12].

This study addressed a problem that is rarely highlighted nowadays: eye impairment in persons affected by leprosy that can occur while having treatment, in patients who experience a reaction to leprosy treatment (indirectly), or after the patients are declared RFT. Therefore, patients often ignore eye complaints because they feel they have completed their treatment.

In this study, we assessed QoL based on the questionnaire from the NEI-VFQ-25, which is usually used to assess QoL related to visual impairment. We aim to investigate whether this questionnaire could be used to assess the quality of life of RFT people with/without disabilities. In addition, the degree of disability, visual acuity, and the identification of possible correlations between these factors among RFT people in Indonesia are the merits of this paper. We recommend using this instrument to monitor and evaluate VR QoL among leprosy patients with visual impairments who have been released from treatment periodically; thus, the treatment can be given immediately to prevent further disability. This instrument can also be applied for patient screening prior to physical examination by an ophthalmologist.

## Methods

A cross-sectional study was conducted in Singkawang, West Kalimantan and Tangerang, Banten, Indonesia between 2018 and 2019. The study was conducted by Cipto Mangunkusumo Hospital’s medical team titled KATAMATAKU (*Identifikasi Tanda-tanda Kelainan Mata, Ekstremitas, dan Kulit pada Kusta*), consisting of ophthalmologists, dermatologists, and medical rehabilitation specialists. The inclusion criteria were all former leprosy patients who had declared RFT and had given consent to participate, completed the VR-QoL questionnaire, and undergone all examination series during the program which included eye, skin, and extremities examinations. The exclusion criteria were leprosy patients who were still on leprosy medication based on the patient's acknowledgment and/or suffering from severe mental disorders or difficulty communicating based on the assessment of the research team onsite. Data were collected by interviewing and examining a total of 363 RFT persons in Singkawang and Tangerang, 325 subjects of whom met the eligibility criteria. Physical examination included the detection of signs and symptoms of leprosy such as abnormalities and deformities in the eyes (visual acuity using Snellen chart), and extremities (deformity, motoric and sensory nerve function).

To assess VR-QoL, we used the National Eye Institute 25-item Visual Function Questionnaire (NEI-VFQ-25) that had been validated and translated into Indonesian. It was administered by the interviewer as a printout at the study site. Subjects who were illiterate were assisted in filling out the questionnaire [13]. To prevent from being bias when assisting patients completing the questionnaire, the interviewers had been trained on how to ask the questions without directing the participants’ answers and to use language understood by the subjects. The selected interviewers were general practitioners and nurses who volunteered to participate in this program. They also received training for filling out the NEI-VFQ-25 questionnaire. We also provided interview guidance that could be learned by the selected interviewers.

The NEI-VFQ-25 targeted question subscales include global vision, difficulty with near vision activities, difficulty with distance vision activities, limitation in social functioning, role limitations due to vision, dependency on others due to vision, mental health symptoms due to vision, driving difficulties, limitations with peripheral, colour vision, and ocular pain [14]. Each subscale in the NEI-VFQ-25 is calculated based on the method that has been described by the developer and can range from 0 to 100, where 0 is the worst and 100 indicates the best related to vision quality of life. Mangione et al. measured quality of life using the NEI-VFQ-25 questionnaire because this questionnaire provides reproducible data and validity, especially if used in various conditions with varying degrees of eye disease severity. This questionnaire is also widely chosen because it is specific [15, 16].

Disability in leprosy is defined by the WHO grading system: Grade 0—absence of disability (no anaesthesia) and no visible damage or deformity on eyes, hands, or feet; Grade 1 − loss of protective sensibility on eyes, hands, and feet; Grade 2—presence of deformities or visible damage to the eyes, hands, or feet. The highest grade of disability of any of these body sites was used as an overall indicator of the disability status of an individual with leprosy. This assessment was used because some of the items in the NEI-VFQ-25 questionnaire not only require a healthy vision but are comprehensive with the extremities (e.g. walking downstairs and driving) [17, 18].

We performed two kinds of visual acuity examinations consisting of Uncorrected Visual Acuity (UCVA) without the use of trial glasses or lenses, and Best-Corrected Visual Acuity (BCVA) using trial lenses. Afterwards, we classified visual acuity into four classifications based on the World Health Organization of Vision Impairment Classification which are:

- 1 = for no or mild visual impairment (visual acuity is ≥ 6/18), ≥ 0.33
- 2 = for moderate visual impairment (visual acuity < 6/18 to ≥ 6/60), ≥ 0.10
- 3 = for severe visual impairment (visual acuity is < 6/60 to ≥ 3/60) and ≥ 0.05
- 4 = for blindness (visual acuity < 3/60 to no light perception) < 0,05 [19]

### Statistical analysis

Statistical analysis was performed using SPSS for Macintosh version 25.0. IBM Corp. Released 2017. IBM SPSS Statistics for Windows, Version 25.0. Armonk, NY: IBM Corp. Data were presented in the form of tables, and narratives. Relationships and comparisons among and between variables were evaluated using ANOVA or Kruskal–Wallis test and Mann–Whitney test, the alpha level was set at *p* < 0.05.

## Results

A total number of 325 subjects, consisting of 76 subjects from Singkawang and 249 from Tangerang, were analysed. Table 1 shows the comparison of subject characteristics in all RFT persons by demographic information. The mean age of the total subjects was 53.21 ± 12.32 years old. Subjects were predominantly males, in a 1.7:1 ratio compared to females. Most of the subjects (49.8%) had non-fixed income (taxi bike drivers, construction workers, stall owners, and freelancers). Nearly half of them were uneducated (47.4%).

**Table 1:** Demographic data of RFT persons, Singkawang and Sitanala Indonesia, 2018–2019

Variables	Total subjects (*n* = 325)	
	N (%)	
**Age (Mean + SD)**	53.21 ± 12.32	
N/A	22	(6.8)
**Gender**		
Male	206	(63.4)
Female	119	(36.6)
**Occupation**		
Fixed income^a^	10	(3.1)
Non-fixed income^b^	162	(49.8)
Unemployed^c^	153	(47.0)
**Education**		
Undergraduate	1	(0.3)
Senior high school	33	(10.2)
Junior high school	26	(8.0)
Primary school	111	(34.2)
Uneducated	154	(47.4)

*Abbreviations*: *N/A* not available, *RFT* Released From Treatment

^a^factory worker, teacher, office employee

^b^taxi bike drivers, construction workers, stall owners, freelancers

^c^students, housewives, not working

As shown in Table 2, based on WHO disability grading, 66 (20.3%) subjects were in grade 0, 11 (3.4%) subjects were in grade 1, while 248 (76.3%) subjects were in grade 2. The majority of the subjects were diagnosed with MultiBacillary (MB) type of leprosy when they were first examined (84.6%), and had a duration of leprosy above 5 years (51.4%). Despite the highest number of subjects being in grade 2 disability, 76.6% of the patients still had no or mild visual impairment. This is due to as many as 54 (16.6%) subjects having grade 2 disability in their eyes, 227 (69.8%) in hands, and 234 (72.2%) in their feet. However, the difference in scores between these groups was not statistically significant (*p* = 0.77).

**Table 2:** Clinical characteristics of RFT persons, Singkawang and Sitanala Indonesia, 2018–2019 based on WHO disability grading

Variables	Total subjects (*n* = 325)		Grade 0 (*n* = 66)		Grade1 (*n* = 11)		Grade 2 (*n* = 248)	
	n	(%)	n	(%)	n	(%)	n	(%)
**Types of leprosy**								
PB	50	15.4	22	33.3	2	18.2	26	10.5
MB	275	84.6	44	66.7	9	81.8	222	89.5
**Duration of leprosy**								
< 2 years	34	10.5	5	7.6	0	0	29	11.7
2–5 years	107	32.9	18	27.3	5	45.5	84	33.9
> 5 years	167	51.4	43	65.2	5	45.5	119	48
N/A	17	5.2	0	0	1	9.1	16	6.5
**UCVA**								
No or mild visual impairment	203	62.5	42	63.6	6	54.5	155	62.5
Moderate visual impairment	91	28	17	25.8	3	27.3	71	28.6
Severe visual impairment	14	4.3	4	6.1	1	9.1	9	3.6
Blindness	17	5.2	3	4.5	1	9.1	13	5.2
**Locations of Disability**								
Eye	325	100	266	81.8	5	1.5	54	16.6
Hand	325	100	65	20	33	10.2	227	69.8
Foot	325	100	52	16	39	12	234	72

*Abbreviations*: *RFT* Released From Treatment, *PB* Paucibacillary, *MB* Multibacillary, *UCVA* Uncorrected visual acuity

Table 3 shows Uncorrected Visual Acuity (UCVA) and Best-Corrected Visual Acuity (BCVA) examination results. More than half of the subjects were classified as having mild or no visual impairment according to the WHO Vision Impairment Classification. We can see that there were improvements in all classifications when the subjects were best-corrected with lenses, so it may indicate that Uncorrected Refractive Error (URE) was the majority cause of visual impairment and blindness in these subjects.

**Table 3:** Distribution of visual impairment in RFT persons, Singkawang and Sitanala Indonesia, 2018–2019 based on WHO classification for visual acuity

Classification	UCVA *n* = 325 (%)		BCVA *n* = 325 (%)	
No or mild visual impairment	203	(62.5)	252	(77.5)
Moderate visual impairment	91	(28.0)	53	(16.3)
Severe visual impairment	14	(4.3)	7	(2.2)
Blindness	17	(5.2)	13	(4.0)

*Abbreviations*: *UCVA* Uncorrected visual acuity, *BCVA* Best Corrected visual acuity

The total quality of life score (composite score) is the average of 10 to 11 subscale scores obtained from the average of the scores for each question in the NEI-VFQ-25 questionnaire in this study. The revised scoring algorithm excludes the single-item general health rating question from the calculation of the vision-targeted composite score. The maximum score in the normal population without impairment is 100 (100%) [20]. The mean total quality of life score in each grade based on WHO disability grading is shown in Table 4. The median total quality of life score for all subjects in grade 0 is 87 (30–100), in grade 1 74.5 (50–98), and in grade 2 70 (13–98), which means that for all subjects, there has been a decrease in quality of life about 13%, 25.5% and 30% of the maximum value in each grade, respectively. These three median differences were statistically significant (*p* < 0.001).

**Table 4:** Comparison between WHO Grade of Disability and VRQoL classification based on NEI-VFQ-25 in RFT persons, Singkawang and Sitanala Indonesia, 2018–2019 (*n* = 325)

NEI-VFQ-25 subscale	WHO Disability Grading^a^			*P* value^b^
	Grade 0	Grade 1	Grade 2	
General Health	25 (0–100)	25 (0–100)	20 (0–100)	0.025
General Vision	60 (0–100)	50 (20–100)	40(0–100)	0.005
Ocular Pain	100(25–100)	62.5(38–100)	75(13–100)	< 0.001
Near Vision	100(50–100)	75(33–100)	67(0–100)	< 0.001
Distance Vision	100(58–100)	83.5(58–100)	75(25–100)	< 0.001
Social Function	100(50–100)	88(50–100)	88(25–100)	< 0.001
Mental Health	94(25–100)	84.5(50–100)	69(0–100)	< 0.001
Role Limitation	100(0–100)	88(50–100)	75(0–100)	< 0.001
Dependency	100(0–100)	100(50–100)	75(0–100)	0.007
Driving^c^	92(0–100)	83(25–100)	75(0–100)	0.070
Color Vision	100 (50–100)	100(50–100)	100(0–100)	< 0.001
Peripheral Vision	100(50–100)	87.5(50–100)	75(0–100)	< 0.001
Composite Score	87(30–100)	74.5(50–98)	70(13–98)	< 0.001

*Abbreviations*: *WHO* World Health Organization, *VRQoL* Vision-related Quality of Life, *NEI-VFQ-25* National Eye Institute Visual Functioning Questionnaire–25

^a^ Median(min–max)

^b^ Kruskall-Walis test

^c^ Among all subjects only (105 patients were driving)

General health denoted the lowest median score in all severity grades among all questions, while colour vision had the highest median score for all severity grades. The median score on the near vision subscale was worse than the distance vision, and this can be caused by the average age of patients in this study, which was over 50 years old. Of the total eleven subscales we analysed, there was a significant relationship between the WHO Disability Grading and the VR-QoL Classification based on the NEI-VFQ-25 (*p* < 0.05). We excluded the driving subscale due to the high missing rate in this population.

Afterward, we assessed each subscales and determined disability grade 0 as our reference because it was considered as not having any disability compared to WHO disability grade 1 and 2. Using the Mann–Whitney test as shown in Table 5, we concluded when VR-QoL compared to grade 0 and grade 1, disability groups were almost entirely not statistically significant, only the ocular pain and near vision subscales were significantly different. In contrast, in the comparison between grade 0 and grade 2 disability, all subscales were statistically significant.

**Table 5:** Comparison between WHO grade of disability and VRQoL classification based on NEI-VFQ-25 using grade 0 of disability as a reference in former leprosy patients, Singkawang and Sitanala Indonesia, 2018–2019 (*n* = 325)

NEI-VFQ-25 subscale	WHO disability grading^a^		
	Grade 0	Grade 1	Grade 2
General health	reference	0.541	0.006
General vision	reference	0.434	0.001
Ocular pain	reference	0.047	< 0.001
Near vision	reference	0.013	< 0.001
Distance vision	reference	0.166	< 0.001
Social function	reference	0.241	< 0.001
Mental health	reference	0.322	< 0.001
Role limitation	reference	0.526	< 0.001
Dependency	reference	0.353	0.002
Driving^b^	reference	0.348	0.023
Color vision	reference	0.549	< 0.001
Peripheral vision	reference	0.084	< 0.001
Composite score	reference	0.137	< 0.001

*Abbreviations*: *WHO* World Health Organization, *VRQoL* Vision-related Quality of Life, *NEI-VFQ-25* National Eye Institute Visual Functioning Questionnaire–25

^a^ Mann–Whitney test

^b^ Among all subjects only (105 patients were driving)

## Discussion

This study evaluates the relationship between vision-related quality of life and the grade of disability among RFT persons in Indonesia. In this study, subjects were predominantly men, and this was similar to the study by Peters et al. in Nigeria which found that the ratio between males to females was 2:1 from a total of 1527 patients [21]. Various studies have reported that the proportion of women affected with leprosy was lower than men. This finding may be due to the underreporting of female leprosy cases. A study from Nepal has stated some factors which were responsible for these under-reported cases, some of which are women with leprosy were illiterate, got married early, had a heavy workload, had poor knowledge, and were unaware of leprosy signs and symptoms. However, in the current study, we have not further analysed the data about women with leprosy in Indonesia; thus, further investigation regarding behavioural characteristics and cultural aspects of female leprosy cases is highly recommended [22].

This study also revealed that the number of uneducated subjects was considerably high with 47% cases and most of the subjects had non-fixed income with 49.8% cases. Our findings are consistent with Mankar et al. study from India which stated the number of illiterate subjects was the highest and most of them were farmers or sedentary workers [10]. We can assume from these conditions that RFT persons will have low health awareness which leads to delayed diagnosis and treatment. Consequently, this delay may lead to an even more severe disability sequel despite the completion of MDT. To increase patient awareness, we can carry out primary prevention activities through education and screening programs. As we have been doing so far in the KATAMATAKU program, the activities consist of preventive, promotive, curative, and rehabilitative aspects for the management of leprosy patients, and during the implementation of the program, we usually collaborate with the community, stakeholders, local government, and academicians. The KATAMATAKU program has also included vision screening and the prescription of glasses for leprosy patients with visual impairment.

Visual impairment in RFT persons revealed that most of the subjects suffered from no or mild visual impairment. It represents 5.2% of blindness in UCVA, but if their visual acuity is corrected with glasses, it improves to 4.0%, while the 28% of the subjects with moderate visual impairment in UCVA can improve to 16% after being corrected with glasses (Table 3). Early visual acuity screening and the use of glasses to reduce visual impairment play an important role in Leprosy surveillance programs, particularly in MB cases where the disability is higher. This finding is similar to a study by Thompson et al. which found that 2.9% patients were blind and 20.7% had a moderate visual impairment [23].

Quality of life has 5-dimensional aspects which include aspects of vision, economic, social, functional aspects (self-care, mobility, activity level, activity of daily living), as well as psychological and emotional aspects (cognitive function, emotional well-being) [24]. In the NEI-VFQ-25 questionnaire, there is a driving subscale which we excluded because there were only 105 (32.3%) subjects who claimed to be able to drive, 35 (10.8%) had to stop driving, while the other 185 (67.7%) were never driving or did not want to answer this section. Of those subjects who were able to drive, only 56 subjects claimed to be able to drive a car, while this section of the questionnaire was specially made for car driving, not other vehicles like motorcycles or bicycles which are common transportation in Indonesia. The possible explanation for this situation was the poor economic state of mostly leprosy patients in Indonesia. A similar possible explanation in other studies from different populations also showed a high percentage of missing values for the ‘Driving’ subscale [25].

Most subjects in this study had almost entered the geriatric age. Leprosy amongst the elderly deserves attention because of the increased susceptibility to having a disability in this age group, with their higher risk of reaction, their greater level of comorbidity, resulting in low quality of life. The same result showed by the study of Bello et al. which found low Health-Related Quality of Life (HRQoL) among their samples, those of whom were the elderly patients affected by leprosy in South Ghana [12]. Okpo et al. also reported in their study in Kano State, Nigeria that the prevalence of low vision and blindness among their subjects was high, which may be due to the subjects they studied experiencing complications of ocular leprosy and/or as part of the age-appropriate degenerative process of the subjects.^25^ Another study in Cameroon also stated that the prevalence of visual disability in leprosy patients was very high, but only one-third of the cases were due to complications of leprosy that they suffered, since most of which were caused by age-related pathological processes [26].

Using grade 0 of WHO disability grading as a reference, nearly all parameters are statistically insignificant between disability grade 1 and the vision-related quality of life. In contrast, the comparison between grade 0 and grade 2 disability showed all subscales were statistically significant that may make daily life activities difficult in the beginning (see Table 5). Regardless of their disability, after a long time of getting used to living with their disability, they were finally able to adapt and carry out daily activities. This result appeared to be similar to the study by Prado et al. in Brazil which concluded that disability grades do influence the physical activity of leprosy patients. There is an assumption that neural involvement in limbs and eyes occurs slowly [27]. They slowly develop new ways of holding objects, allowing them to perform routine activities independently. Our study also shows that most of the subjects suffering from leprosy for more than 5 years 167 (51.4%) had adapted to their limitations. On the other hand, loss of protective sensation increases the risk of injury and can result in loss of integrity of the affected organ resulting in new disability.

Leprosy mainly affects peripheral nerves in the extremities and eyes. In our report, subjects who were uneducated and had non-fixed income were the highest number among others; thus, education and individual skills are two substantial points for subjects to get a better job and higher income. Negative stigma from the community towards people affected by leprosy is assumed to highly influence the opportunity of leprosy patients from obtaining decent employment due to their disabilities. Therefore, the stigma regarding leprosy patients that still persists in the community should be removed, so they can be accepted well in their surroundings and have a suitable chance for work. To conclude, the use of the NEI-VFQ-25 questionnaire that has been analysed in this study can be beneficial for the improvement of clinical management in leprosy patients in terms of initial screening in primary health care settings and monitoring of leprosy patients to prevent further disability and improve QoL of the patients.

The limitation of this study is the biased sample, as non-random samples from a settlement of elderly former leprosy patients near Jakarta and Singkawang were used, and the cross-sectional study design. Furthermore, we suggest that the NEI-VFQ-25 questionnaire needs to be modified, especially the driving sub-scales, because these questions were not applicable, especially in low economic settings.

## Conclusion

In conclusion, in regards to the VR-QoL assessment compared with disability grading, it is noteworthy that the disabilities of the eye, hand, and foot affect their VR-QoL. Subjective results based on the questionnaire were in concordance with objective results from the examination.

The NEI-VFQ-25 seems applicable to measure the VR-QoL of RFT persons with leprosy cases and can be used as an initial screening in primary health care settings to increase awareness of their disability before a thorough physical examination. We hope that eye health services can be expanded to these people so that their vision and consequently their VR-QoL can be improved with a pair of glasses or cataract surgery. Further research with a larger number of subjects is still needed, especially in more endemic locations.

## Data Availability

The datasets analysed during the current study are not publicly available but are available from the corresponding author upon reasonable request.

## Abbreviations

BCVA: Best-Corrected Visual Acuity
KATAMATAKU: Identifikasi Tanda-tanda Kelainan Mata, Ekstremitas, dan Kulit pada Kusta
MB: MultiBacillary
N/A: Not Available
NEI-VFQ-25: National Eye Institute Visual Functioning Questionnaire–25
PB: PauciBacillary
QoL: Quality of Life
RFT: Release From Treatment
UCVA: UnCorrected Visual Acuity
VR-QoL: Vision-Related Quality of Life
WHO: World Health Organization
